# c-Abl Kinase Is a Regulator of α_v_β_3_ Integrin Mediated Melanoma A375 Cell Migration

**DOI:** 10.1371/journal.pone.0066108

**Published:** 2013-06-21

**Authors:** Chunmei Zhang, Chao Yang, Ruifei Wang, Yang Jiao, Khamal Kwesi Ampah, Xiaoguang Wang, Xianlu Zeng

**Affiliations:** 1 Institute of Genetics and Cytology, Northeast Normal University, Changchun, Jilin Province, China; 2 Department of Cell Biology, Norman Bethune College of Medicine, Jilin University, Changchun, Jilin Province, China; 3 Department of Bioscience, Changchun Teachers College, Changchun, Jilin Province, China; China Medical University, Taiwan

## Abstract

Integrins are heterodimeric transmembrane receptors that physically link the extracellular matrix (ECM) to the intracellular actin cytoskeleton, and are also signaling molecules that transduce signals bi-directionally across the plasma membrane. Integrin regulation is essential for tumor cell migration in response to growth factors. c-Abl kinase is a nonreceptor tyrosine kinase and is critical for signaling transduction from various receptors. Here we show that c-Abl kinase is involved in A375 cell migration mediated by α_v_β_3_ integrin in response to PDGF stimulation. c-Abl kinase colocalizes with α_v_β_3_ integrin dynamically and affects α_v_β_3_ integrin affinity by regulating its cluster. The interaction between c-Abl kinase and α_v_β_3_ integrin was dependent on the activity of c-Abl kinase induced by PDGF stimulation, but was not dependent on the binding of α_v_β_3_ integrin with its ligands, suggesting that c-Abl kinase is not involved in the outside-in signaling of α_v_β_3_ integrin. Talin head domain was required for the interaction between c-Abl kinase and α_v_β_3_ integrin, and the SH3 domain of c-Abl kinase was involved in its interaction with talin and α_v_β_3_ integrin. Taken together, we have uncovered a novel and critical role of c-Abl kinase in α_v_β_3_ integrin mediated melanoma cell migration.

## Introduction

The metastatic sequence of tumor cells is understood to involve detachment of cell within primary tumor, local migration and intravasating into the bloodstream, and extravasating into tissue, further local crawling, migration and invasion, generation of new colonies. Migration is a critical process for tumor cell to overcome this remarkable set of challenges [1]–[3]. Cell migration is a highly complex and regulated process, in which intracellular and extra cellular signals conjoin to produce a coordinated response. The direction of cell migration is controlled by growth factors and ECM gradients. Cells respond to local activation and amplification of signaling events on the side facing the attractant, which results in the orderly rearrangement of adhesive structures that connect the cell to the ECM [4], [5]. There are several adhesion receptor families involved in the migration of cells, the best-studied adhesion receptors, and of particular interest in migration, are integrins. Integrins, the heterodimers consisting of α and β subunits, contribute in multiple ways to the process of cell migration. First, integrin form connection between the intracellular actin cytoskeleton and the ECM, which is critical for many cellular processes including efficient cell movement besides providing structural support for cells [6]. Second, integrins also mediate signal transduction. They mediate signal transduction through the cell membrane in both directions: binding of ligands to integrins transmits signals into the cell and results in cytoskeletal re-organization, gene expression and cellular differentiation (outside-in signaling); on the other side, signals from within the cell (in response to local stimuli) can also propagate through integrins and regulate integrin ligand-binding affinity and cell adhesion (inside-out signaling) [7], [8]. This bidirectional signaling is mainly mediated by the short cytoplasmic tails of the two integrin subunits [9]. Integrin α_v_β_3_ is known to be responsible for cell attachment and spreading, as well as cell locomotion. The expression of integrin α_v_β_3_ has been detected in different types of tumor cells, including breast, prostate, ovary, melanomas and gliomas, this expression has been reported to correlate with an aggressive phenotype and metastatic dissemination. Specifically, the increase of migration in tumor cell is due in part to integrin α_v_β_3_ [10], [11].

Cytoplasmic tyrosine kinases have been demonstrated to be critical in integrin signaling, such as Src-family kinases and focal adhesion kinase (FAK) [7]. c-Abl, a non-receptor tyrosine kinase, localized both in the nucleus and cytoplasm, plays an essential role in signaling transduction of various receptors and is involved in the regulation of cell growth, survival and morphogenesis [12]. c-Abl proteins are characterized by a unique N-terminus followed by a SH3 domain, a SH2 domain and the catalytic core. SH2 and SH3 domains are involved in protein-protein interactions and also regulate the kinase activity [13]. Additionally, the C-terminus includes F-(filamentous) and G-(globular) actin-binding domains, NESs (nuclear export sequences) and proline-rich sequences with an affinity for SH3-containing proteins. c-Abl also contains NLSs (nuclear localization signals) and DNA-binding sequences which are important for nuclear functions [14]–[16].

The mutant forms of c-Abl gene are well known to be involved in hematopoietic malignancies such as chronic myeloid leukemia (CML). To date, extensive evidence concerning the role of c-Abl kinase in the integrin signaling transduction has been accumulated. Earlier reports indicated that integrin can regulate c-Abl kinase activity and cytoplasmic-nuclear transport in fibroblastic cells adhering to fibronectin [17], meanwhile, c-Abl kinase contributes to the activation of MAPK in cells plated to fibronectin [18]. Furthermore, c-Abl is a key intracellular molecule-mediating angiogenesis induced by bFGF which associates with β_3_ integrin, and c-Abl can mediate endothelial cells apoptosis when integrins α_v_β_3_ and α_v_β_5_ were inhibited [19], [20]. Adhesion-activated c-Abl kinase phosphorylates cytoskeletal protein paxillin, which is a component of focal adhesion [21]. In addition, c-Abl protein levels, as assessed by immunohistochemistry, are increased in many solid tumors, but the increased expression is not consistently correlated with disease grade [22], [23]. Some other findings demonstrated that the activated c-Abl kinase may play a role in some solid tumors, such as non-small cell lung cancer (NSCLC) and in aggressive human breast cancer [24], [25]. Only more recently, it was reported that c-Abl kinase promotes melanoma cell invasion and drives metastatic progression via inducing transcriptional upregulation and activation of MMPs [26]. However, little is known about the role of c-Abl kinase in integrin signaling of solid tumor. These data prompted us to examine whether c-Abl kinase can interact with integrin to play a role in solid tumor progression.

In the present study, we have explored the role of c-Abl kinase in regulating α_v_β_3_ integrin signaling in the migration of melanoma cells. We demonstrated that c-Abl kinase was involved in α_v_β_3_ integrin mediated A375 cell migration induced by PDGF. Endogenous c-Abl kinase and α_v_β_3_ integrin co-localized dynamically in response to extracellular PDGF stimulation, and PDGF stimulation also enhanced α_v_β_3_ integrin binding to its ligands. c-Abl kinase interacted with α_v_β_3_ integrin by using its SH3 domain, and this interaction was dependent on c-Abl kinase activity but not related to F-actin or ligand-integrin binding. The head domain of talin protein played a critical role in this interaction of c-Abl kinase with α_v_β_3_ integrin, and the activated c-Abl kinase directly linked to talin head to regulate integrin activity. Therefore, our work has defined a novel role for c-Abl kinase as a regulator of inside-out integrin signaling in melanoma cell and supports the development of therapeutic interventions geared towards inhibiting c-Abl kinase in the regulation of metastasis in melanoma patients.

## Materials and Methods

### Cell culture and plasmids

Human melanoma A375 cells and human prostate carcinoma DU-145 cells were purchased from the Cell Bank of Type Culture Collection of Chinese Academy of Science (Shanghai, China). All of the cells were cultured in DMEM (Invitrogen) supplemented with 10% heat-inactivated fetal bovine serum (FBS), 100U penicillin and 100 µg/ml streptomycin at 37°C in the presence of 5% CO_2_. A375 and DU-145 cells were detached with trypsin-EDTA (0.05% Trypsin).

The GST-CrkII-C-terminal domain (CTD) plasmid was provided by Dr. Giorgio Scita (European Institute of Oncology, Milan, Italy). The GST-talin2-head domain was kindly given by Dr. Pietro De Camilli (Yale University, School of Medicine, New Haven, CT). The pCMV-human-c-Abl was provided by Dr. Thomas Rudel (Max Planck Institute for Infection Biology, Berlin Germany) and the sequences encoding the domains of c-Abl were amplified by PCR using the expression vector as templates. *Eco*RI and *Xho*I restriction sites were introduced by the PCR primers, and the fragments were subcloned into pGEX vector. pET28-c-Abl-SH3 was constructed as described above. The mutants of tyrosine sites in the SH3 domain of c-Abl kinase were introduced using the specific PCR primers.

### Antibodies and reagents

LM609 (anti-α_v_β_3_ blocking mAb) and TDM29 (anti-β_1_ blocking Ab) were purchased from Millipore; TA205 (anti-talin mAb) was from Serotec; K12 (anti-c-Abl polyclonal Ab, rabbit IgG, sc-131) was purchased from Santa Cruz Biotechnology; Human PDGF-BB was purchased from R&D systems; ECL Plus western blotting detection reagents (RPN2132) and glutathione Sepharose 4B (17-0756-01) were purchased from Amersham Biosciences. G7781 (anti-glutathione S-transferase antibody, rabbit IgG), Cytochalasin D (CD) and PY20 (p-Tyr Antibody) as well as other chemicals were from Sigma-Aldrich. STI571 (Gleevec, Imatinib. Inhibitor of c-Abl kinase) was from Novartis (Basel, Switzerland); Rhodamine-conjugated phalloidin was purchased from Molecular Probes, and Calcein-Am was purchased from invitrogen.

### In vitro protein binding assay

*E. coli* BL21 transformed with pET28-talin2-head was grown and induced with isopropyl β-D-thiogalactoside. His-talin2-head was purified with Ni^2+^Sepharose (Qiagen) according to the manufacturer's instructions. GST or GST fusion protein was eluted from glutathione-Sepharose 4B beads. Approximately 1 µg of His-talin2-head immobilized to 10 µl of Ni^2+^Sepharose was incubated with 0.5 µg of GST or GST fusion protein in 500 µl of modified GBT buffer (10% glycerol, 50 mM HEPES-NaOH (pH 8.0), 170 mM KCl, 7.5 mM MgCl_2_, 0.1 mM EDTA, 1 mM DTT, 1% Triton X-100) containing 1% BSA. After 2 h, the resins were extensively washed with GBT buffer, and the bound proteins were separated by 12% SDS-PAGE and immunoblotted with anti-GST antibody.

### Far-western blot analysis

SDS-PAGE and western blotting were performed as described previously with little modifications. Briefly, immunoprecipitates from A375 cells were separated by 10% SDS-PAGE under reducing conditions and then transferred onto polyvinylidene difluoride (PVDF) membranes (Millipore, Bedford, MA, USA). After nonspecific binding was blocked with PBS containing 5% nonfat milk, the blots were incubated with blocking buffer containing 10 µg/ml GST or the indicated GST-fusion proteins at 4°C overnight. Bound proteins were detected by ECL detection. [27].

### Flow cytometric assay

A375 cells were washed twice and resuspended in PBS. To identify the molecules on cell surface, A375 cells were incubated with isotype IgG and monoclonal antibody (LM609 or TDM29) respectively. After incubation with isotype IgG, LM609 or TDM29, the cells were stained with FITC-conjugated goat anti-mouse IgG (2 µg/ml) for 30 min at 22°C. The labeled cells were detected by a FACScan (Beckman-Counter, USA).

### RNA interference

siRNA targeting c-Abl kinase (5′-GAAGGGAGGUGUACCAUUtt-3′) was synthesized by Shanghai GenePharma, and then was transfected into A375 cells using Lipofectamine 2000 according to the manufacturers' protocols. The extent of suppression and specificity for c-Abl were evaluated by western blotting with anti-c-Abl antibody, anti-actin antibody was used as control.

### In vitro kinase assay

A375 cells were serum starved overnight, and then stimulated and lysed. The lysates were incubated with anti-c-Abl antibody (K12). After 2 h, 20 µl of protein A-Sepharose beads (50% slurry) was added to the antibody/lysates mixture. After 1 h, the immunoprecipitates were washed at least three times with lysis buffer, and then washed three times with the kinase buffer (25 mM Tris, pH 7.5, 2 mM DTT, 5 mM β-glycerophosphate, 1 mM Na_3_VO_4_, 10 mM MgCl_2_). After 5 min preincubation at 30°C, 30 µl of reactions were initiated by adding 3 µg of GST-CrKII-CTD and 5 µM ATP. After 30 min, reactions were terminated by adding 20 µl of 3×SDS sample buffer and resolved by SDS-PAGE.

### Protein expression and GST pull-down assay

Production of GST and GST-fusion proteins was induced in E.coli strain BL-21 transformed with corresponding plasmids by adding 0.3 mM isopropyl β-D thiogalactoside at 37°C for 3 h. Fusion proteins were purified by using glutathione-Sepharose 4B beads according to the manufacturer's instructions. The isolated proteins were stored at 4°C no more than 1 week for experiments.

A375 cells were stimulated and lysed as mentioned above. Cell lysates were incubated with 10 µl of glutathione-Sepharose beads coated with GST or GST fusion proteins. After 2 h, the beads were collected by centrifugation and washed four times with lysis buffer. The bound proteins were eluted by boiling the beads in SDS sample buffer and analyzed by SDS-PAGE.

### Cell migration assay

Wound healing assay: twelve-well plates were coated with 5 µg/ml fibronectin (FN) at 37°C for 1 h, followed by blocking with 1% BSA for 1 h at 37°C. Transfected or control A375 cells were plated in serum-free DMEM until the cells formed a confluent monolayer. Then the cells were serum starved overnight and monolayers were wounded with pipette tip. After washing with PBS for two times, cells were incubated with DMEM containing 20 ng/ml recombinant human PDGF for indicated times and then imaged under phase-contrast microscope (Nikon).

Transwell plates assay: cell migration experiment was performed in transwell plates (8 µm pore size). In brief, the overnight serum starved cells (2.0×10^4^ cells) suspended in 150 µl of DMEM containing 1% fetal bovine serum and antibody or inhibitor were added to the upper chamber, which was precoated with fibronectin on the lower side of the membrane. The lower well was filled with 500 µl of DMEM containing 20 ng/ml recombinant human PDGF (R&D Systems). Cells were allowed to migrate for 8–12 h at 37°C. After migration, the cells on the upper side of the membrane were removed, and the migrated cells on the lower side of the membrane were fixed with methanol, stained with Crystal Violet and dried. The average number of migrated cells in five randomly chosen fields per well was taken to quantify the extent of migration. In addition, each set of experiments was performed in triplicate. Cells were imaged by phase-contrast microscopy.

Three dimensional migration in the agarose drop assay: twelve-well plates were coated with fibronectin (5 µg/ml) at 37°C for 1 h, followed by adding serum-free DMEM medium containing 20 ng/ml recombinant human PDGF, STI571 with different concentrations and 0.3% low melting point agarose (*Invitrogen*), and then placed at 4°C for 25 min to allow the agarose to solidify. Cells were trypsinized and 3 µl centrifugal deposits were dropped into the middle of the solid medium. Cells drops were imaged by phase-contrast microscopy. The cell migration distance was detected by measuring the semidiameter.

### Cell adhesion assay

The adhesion assay was performed as previously described. Briefly, 48-well plates were coated with fibronectin (5 µg/ml) at 37°C for 1 h, and then blocked with 1% BSA for 1 h at 37°C. Overnight serum starved cells were resuspended in DMEM containing 20 ng/ml PDGF, and antibody or inhibitor remained in the medium throughout the assay period. Control cells were not stimulated with PDGF or wells were not coated with fibronectin. Cells were allowed to adhere at 37°C for different time. After incubation, unbound cells were removed by washing three times with PBS, and the adherent cells were fixed with 4% paraformaldehyde at room temperature. The numbers of firmly adherent cells were imaged by phase-contrast microscopy and then quantitated.

### Immunoprecipitation and immunoblotting

Overnight serum starved A375 cells were trypsinized, washed with PBS and suspended in DMEM at a density of 10^7^ cells/ml. For treatment, cells were divided into aliquots of 1 ml, and treated with PDGF or inhibitors as indicated. Cells were incubated for the required time ranging from 5 to 30 min, the stimulation of cells was terminated by placing the plates or tubes on ice. Then the treated cells were washed with ice-cold PBS and lysed in RIPA buffer (50 mM Tris-HCl, pH 7.6, 500 mM NaCl, 1% Triton X-100, 0.5 mM MgCl2, 1 mM PMSF, 10 μg/ml leupeptin, 10 μg/ml aprotinin). After incubation on ice for 15 min, the lysates were centrifuged at 14,000 *g* for 30 min. The supernatants were incubated with the indicated antibodies at 4°C for 2 h, and then 20 µl of protein A/G-Sepharose beads (50% slurry) was added. After incubation for another 1 h at 4°C, the immune complexes were washed three times with lysis buffer and resolved by SDS-PAGE. After protein transfer, the nitrocellulose membranes were incubated with 5% nonfat milk in TBST (20 mM Tris-Hcl, pH 7.5, 50 mM NaCl, 0.05% Tween 20), and then with the indicated primary antibodies and the HRP-conjugated secondary antibodies at 37°C for 1 h, respectively. Chemiluminescent detection was performed by using ECL Plus western blotting reagents.

### Immunofluorescence microscopy

A375 cells were cultured on fibronectin-coated coverslips. After serum starvation for 3 h, cells were stimulated with PDGF for required time. In inhibition experiments, cells were preincubated with STI571 for 10 min, and the inhibitor remained in the medium throughout the assay. The cells were subsequently washed twice with PBS and then fixed for 10 min at room temperature with 4% paraformaldehyde. After washing with PBS, cells were incubated for 1 h at room temperature with primary antibody followed by secondary antibody conjugated with either Alexa Fluor 488 or Alexa Fluor 647 (Invitrogen). All of these stained cells were observed under a confocal microscope.

### Statistical analysis

The experiments were repeated at least 3 times. Results are expressed as mean ± SD. and differences between means were determined by one-way analysis of variance. *P* value less than 0.05 was considered statistically significant.

## Results

### PDGF induced melanoma cell migration is α_v_β_3_ integrin dependent

**Figure 1:**
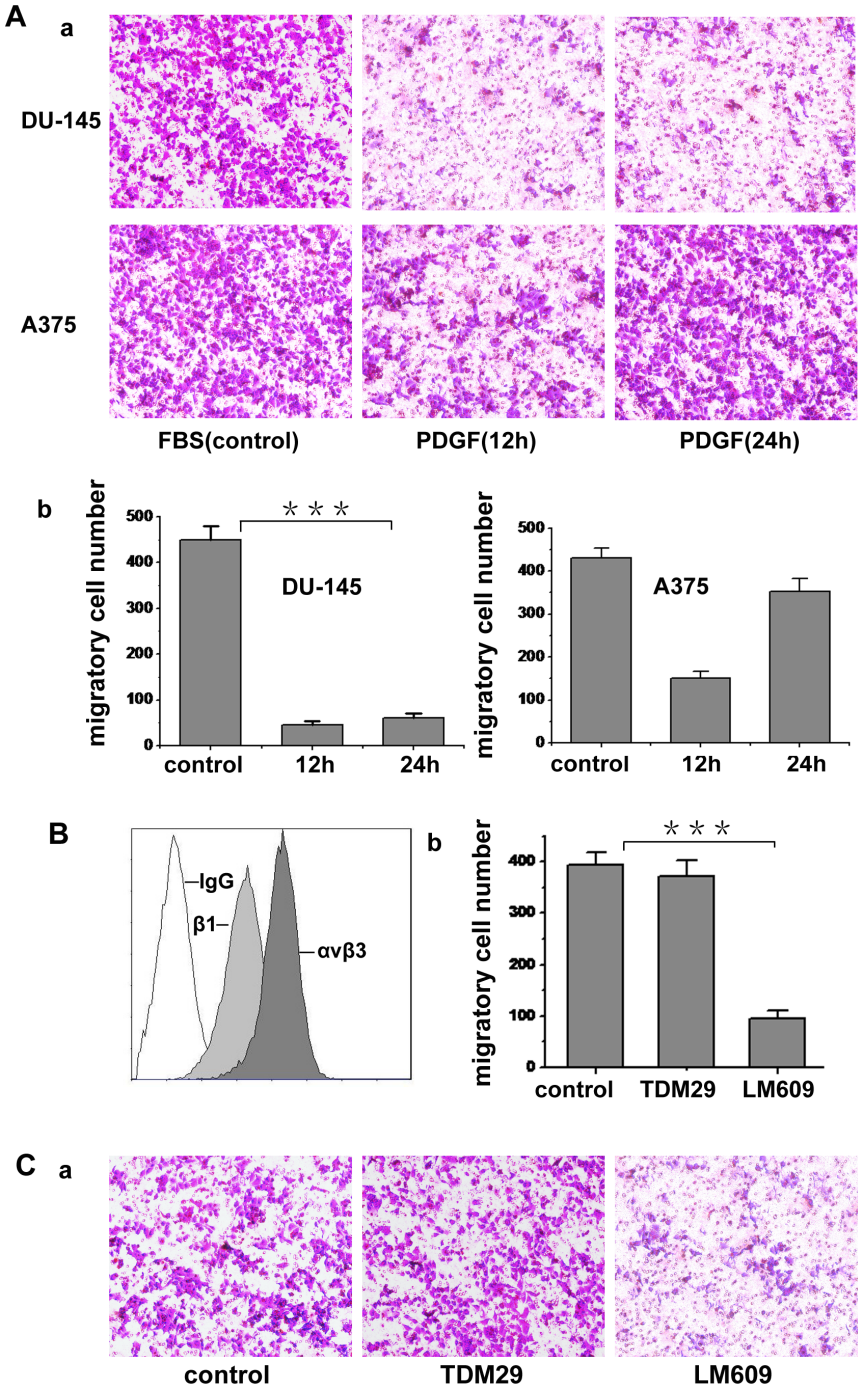
PDGF induces tumor cell migration. (A) The cell suspension containing 0.05% serum was seeded onto 0.8 μm diameter upper transwell chamber, which was coated with fibronectin. The lower chamber contained 3% serum (FBS) or 50 ng/ml PDGF to induce cell migration. Cells were cultured for 24 h in each chamber. The migrating cells were fixed and stained with crystal violet and photographed. The cell migration was determined by counting cells in randomly selected five microscopic field per well. Bars represent mean ± S.D. (b was statistic value of a). (B) Expression of α_v_β_3_ and β_1_ integrin on A375 cell surface was evaluated by LM609 (blocking anti-α_v_β_3_ integrin antibody) and TDM29 (specific anti-β_1_ integrin antibody). IgG was used as negative control. (C) The cell suspension containing 0.05% serum and antibody LM609 or TDM29 (50 µg/ml) was put into 0.8 μm diameter upper transwell chamber, which was coated with fibronectin. Free-serum medium with 50 ng/ml PDGF was put into the lower chamber to induce cell migration. Cells were cultured for 24 h in the chambers and the migrated cells were fixed and stained with crystal violet and photographed (b was statistic value of a). Cell migration was determined by counting cells in randomly selected five microscopic field per well. Bars represent mean ± S.D of three independent experiments. ***, p<0.001 with respect to the control.

To characterize the function of c-Abl tyrosine kinase in tumor cell migration, two kinds of malignant tumor cells, human melanoma A375 cells and human prostate carcinoma DU-145 cells, were chosen in our experiments. We first examined the protein level of endogenous c-Abl kinase in these cells. The results showed that both A375 cell and DU-145 cell express c-Abl (data not shown). It is documented that cytoplasmic c-Abl kinase can be activated by various growth factors, such as PDGF, EGF, TGF-β and AT-1. Because the PDGF could regulate lamellipodia formation and cell migration, and c-Abl kinase has a functional role in the morphological response to PDGF [13], we chose PDGF-BB as the migration inducer. To investigate the effect of PDGF on tumor cell migration, we treated the two kinds of tumor cells with 20 ng/ml PDGF for 12 h. Fig. 1A shows the migratory potential of these two cell lines in respond to PDGF. Compared with fetal bovine serum, PDGF specifically induced A375 cell migration but not DU-145 cell, so A375 cell was used in the following experiments. Since integrins have crucial role in cell migration, we subsequently tested the expression of integrin α_v_β_3_ and β_1_ on A375 cells. As shown in Fig. 1B, both α_v_β_3_ and β_1_ integrins were expressed on A375 cell surface. To determine the roles of these two integrins in the migration induced by PDGF, α_v_β_3_ and β_1_ were respectively blocked with antibodies in the migration assay. As shown in Fig. 1C, LM609 (blocking anti-α_v_β_3_ antibody) could dramatically inhibit the migration, whereas TDM29 (β_1_-blocking antibody) had no obvious inhibitory effect on the migration, indicating that A375 cell migration induced by PDGF is mainly mediated by integrin α_v_β_3_.

### c-Abl kinase is required for melanoma cell migration induced by PDGF

**Figure 2:**
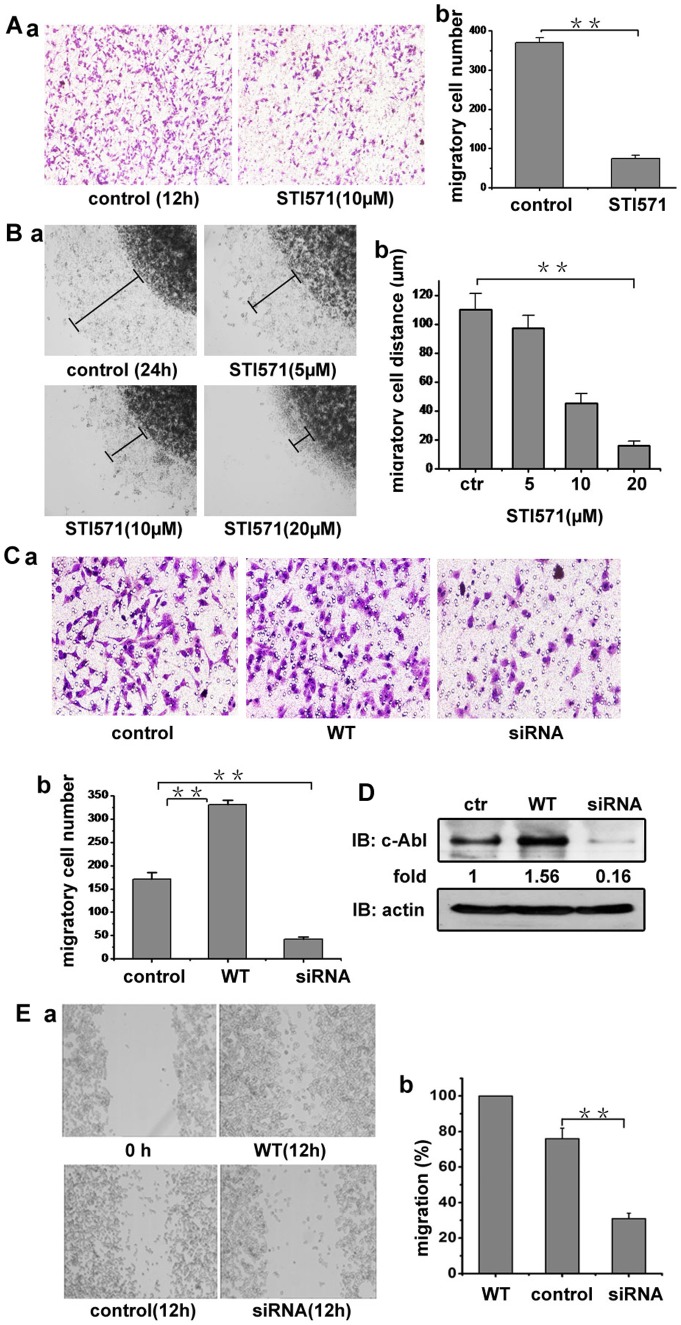
c-Abl kinase affects PDGF induced A375 migration. (A) a, the serum-starved cell suspension, with or without 10 µM STI571 (c-Abl kinase inhibitor), was seeded onto 0.8 μm diameter upper transwell chamber, which was coated with fibronectin. Free-serum medium with 50 ng/ml PDGF was put into the lower chamber to induce cell migration. b, migrations were determined by counting cells in randomly selected five microscope field per well. Bars represent mean ± S.D. (B) the effects of different concentration of STI571 on cell migration. The migration experiment of A375 cells out of agarose drop explants treated with or without STI571 was conducted. After 24 h, the distant of migration was measured using inverted microscope fitted with a rule in eyepiece. a, Cell migration rate was evaluated by the distance of the leading edge of migrating cells from the edge of agarose droplet. b, The distance of cell migration was measured, the extent of cell migration within the drop  =  [(total area/drop area) ×100] –100. (C) A375 cells were transfected with WT c-Abl expression vector or specific siRNA targeting c-Abl. The migration experiment of A375 cell or tansfected cells was performed in transwell plates (b was the statistic value of a). (D) A375 cells or cells transfected with WT c-Abl or c-Abl specific siRNA were lysed (For the transfected cells, cells were lysed after 24 h transfection). Lysates were blotted with c-Abl and actin antibodies. (E) a. A375 cells or the cells transfected with WT c-Abl or c-Abl specific siRNA were plated into 24-well plated and allowed to form a confluent monolayer. After overnight serum starvation, the cell monolayer was “wounded” with a pipette tip. The cells were incubated with DMEM containing 20 ng/ml PDGF and then imaged. b. Cell migration was determined as shrinkage of an average gap area. Bars represent mean ± S.D of three independent experiments. **, p<0.01, ***, p<0.001 with respect to control.

We next explored whether c-Abl kinase is involved in the A375 cell migration induced by PDGF based on the above results that PDGF can induce A375 cell migration and the accumulating evidences that PDGF can regulate c-Abl kinase activity. As shown in Fig. 2A, c-Abl kinase inhibitor STI571 (10 µM) substantially inhibited the PDGF induced A375 cell migration. Furthermore, by testing the migration in 3D agarose drop model, STI571 was shown to reduce the α_v_β_3_-mediated A375 cell migration in a dose dependent manner (Fig. 2B). To confirm the role of c-Abl kinase in melanoma cell migration triggered by PDGF, WT-c-Abl kinase expression vector or specific siRNA targeting c-Abl kinase was transfected into A375 cells. In migration assay, the overexpression of wild type c-Abl kinase increased A375 cell migration and siRNA-mediated c-Abl kinase knockdown effectively inhibited the cell migration, compared with the non-transfected A375 cells (Fig. 2C). The c-Abl kinase protein level in the transfected A375 cells are shown in Fig. 2D. Similar results were obtained from the wound healing assay. As shown in Fig. 2E, the c-Abl-transfected cells migrated to fill about most of the wounded area within 12 h, while c-Abl knockdown cells showed little migration compared with the control cells, indicating that c-Abl kinase is involved in the melanoma cell migration induced by PDGF.

### c-Abl kinase colocalizes with α_v_β_3_ integrin and potentiates α_v_β_3_ integrin mediated A375 cell adhesion

**Figure 3:**
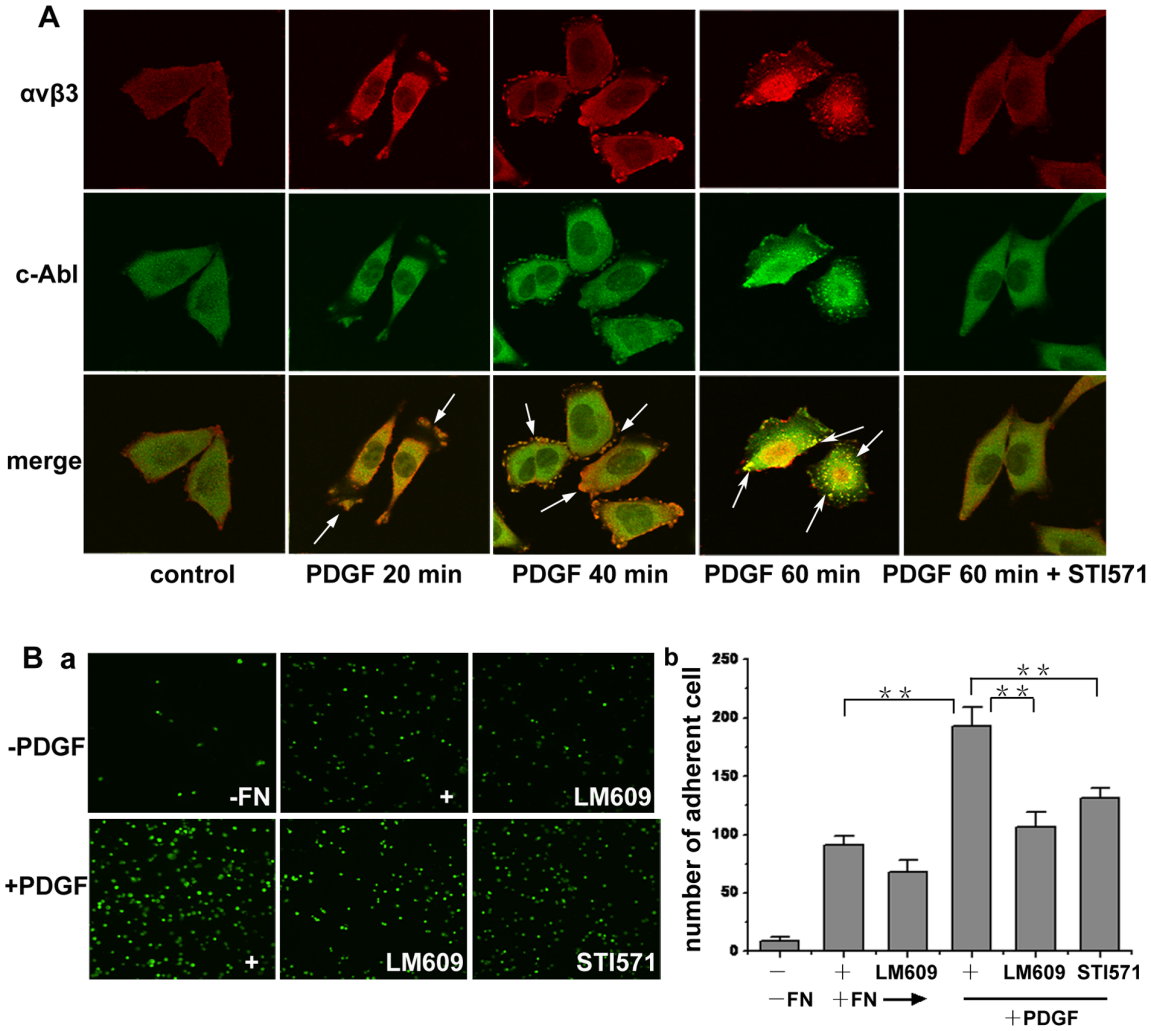
c-Abl kinase mediates α_v_β_3_ integrin engagement after PDGF stimulation. (A) A375 cells were serum starved for 12 h during spreading, then cells were either untreated or treated with 20 ng/ml PDGF for the indicated times. Cells were fixed, labeled with the antibodies of c-Abl kinase and α_v_β_3_ integrin and imaged under confocal microscope. In inhibiting experiments, cells were preincubated with STI571 for 10 min, and the inhibitor remained in the medium throughout the assay period. (B) serum-starved A375 cells were digested with EDTA and suspended in serum-free medium for 2 h to restore the state of the cells and then stained with Calcein-Am. Then the cells were resuspended in DMEM containing 20 ng/ml PDGF, antibody or inhibitor. Cells were allowed to adhere at 37°C for 1 h. Then the unbound cells were removed, and the adherent cells were fixed and imaged by phase-contrast microscopy (a) and then quantitated (b). Controls were the cells not stimulated with PDGF or the wells not coated with fibronectin. Images were obtained from at least three separate random fields of view. Bars represent mean ± S.D of three independent experiments. **, p<0.01 with respect to control.

The above results imply that c-Abl kinase may has some relations with integrin because integrin plays a crucial role in cell migration, pseudopod formation, spreading and adhesion [6]. We next investigated the relationship between c-Abl kinase and α_v_β_3_ integrin. The subcellular localization of c-Abl kinase and α_v_β_3_ integrin was examined in A375 cells. As shown in Fig. 3A, c-Abl kinase and α_v_β_3_ integrin were dynamically associated with each other in serum-starved A375 cells in response to PDGF stimulation. At the early stage of stimulation (20 min), c-Abl kinase and α_v_β_3_ integrin were mainly situated at the lamellipodia of the spreading cell. Then (40 min), with the cell shape changing during spread, c-Abl kinase and α_v_β_3_ integrin both localized to the focal contacts at the edges of the cells. When cells were stimulated for 1 h, c-Abl kinase and α_v_β_3_ integrin colocalized to form clustered spots, which then distributed in the plasma. The above phenomena were not observed when the cells were pretreated with STI571 before PDGF stimulation, suggesting a possible association of endogenous c-Abl kinase and α_v_β_3_ integrin based on c-Abl kinase activity. Integrin clustering can enhance the binding of integrin to its ligands [9]. To confirm whether α_v_β_3_ integrin cluster after PDGF stimulation and the role of c-Abl kinase in regulating the affinity of α_v_β_3_ integrin, we performed cell adhesion assay. As shown in Fig. 3B, α_v_β_3_ integrin antibody LM609 could inhibit the adhesion, indicating that the adhesion was mediated by α_v_β_3_ integrin. PDGF stimulation led to an increase in the adhesion of A375 cells on the fibronectin, and STI571 inhibited the adhesion dramatically. These results indicate that the increase in the adhesion of A375 cells in response to PDGF stimulation is dependent upon α_v_β_3_ integrin engagement and c-Abl kinase is responsible for the engagement.

### c-Abl kinase interacts with α_v_β_3_ integrin in response to PDGF

**Figure 4:**
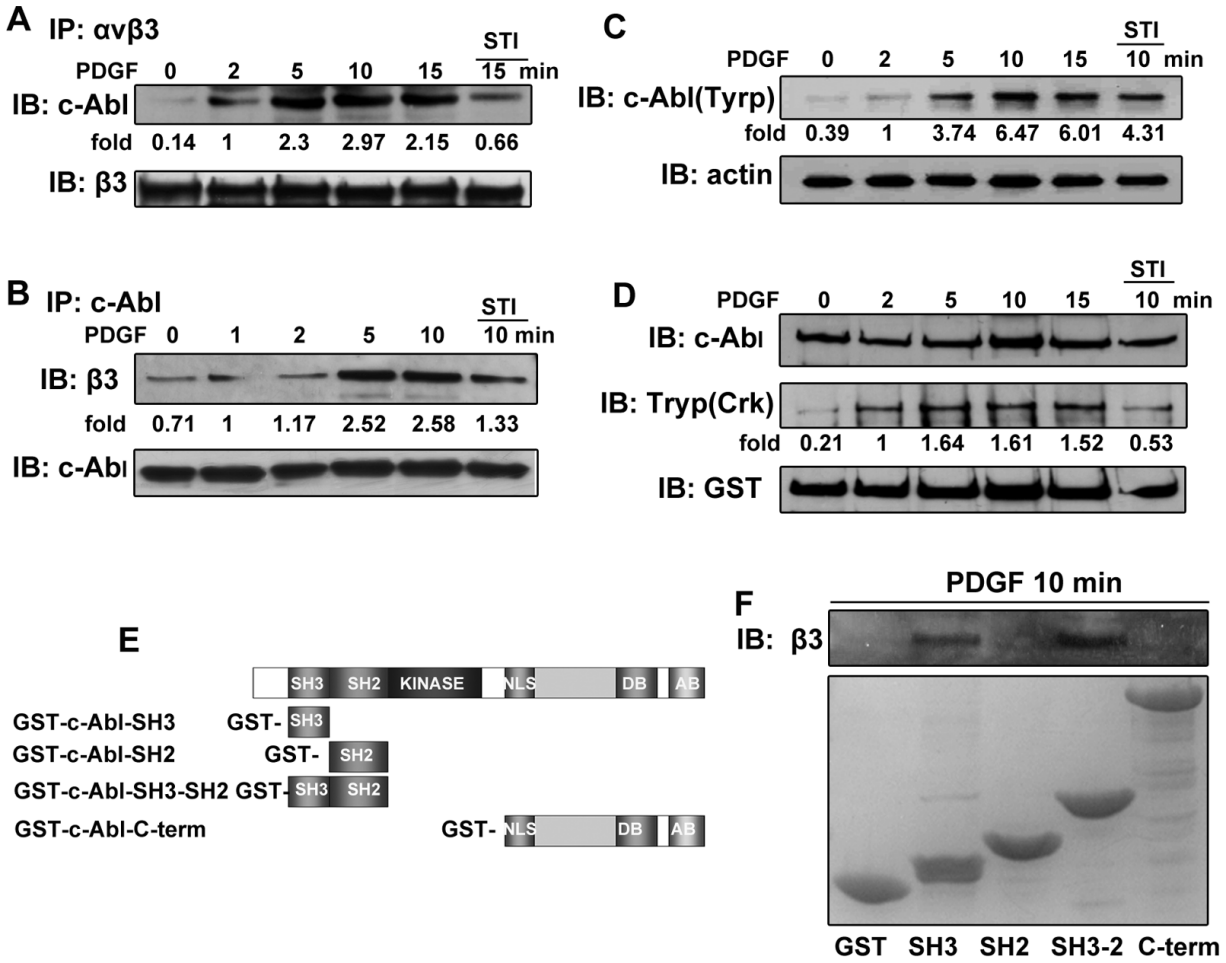
c-Abl kinase activity is required for the interaction of c-Abl and α_v_β_3_ integrin. (A and B) Overnight serum starved A375 cells were trypsinized, washed with PBS and untreated or treated with 20 ng/ml PDGF for the indicated times. Thereafter, the cells were lysed, and the lysates were immunoprecipitated with indicated antibodies and subjected to immunoblotting analysis with the appropriate antibodies. For inhibition assay, cells were pretreated with STI571 for 10 min and the inhibitor remained in the medium throughout the assay. (C) A375 cells were serum-starved and stimulated with PDGF for the indicated times. The lysates of cells were immunoblotted, and the activity of c-Abl tyrosine kinase was tested by immunoblotting with PY20. The loading control was immunoblotted with anti-actin antibody. (D) A375 cells were serum-starved and stimulated with PDGF for the indicated times. The activity of c-Abl kinase was determined by using an in vitro kinase assay with GST-CrkII-CTD as a substrate. Phosphorylation of GST-CrkII-CTD was tested by immunoblotting with PY20 (p-Tyr Antibody). The level of GST-CrkII-CTD was detected with anti-GST antibody to demonstrate equal loading. (E) A schematic diagram of GST c-Abl domains. (F) A375 cells were serum-starved and stimulated with PDGF for 10 minutes. The GST c-Abl domain pull-down assay was done, and the bound proteins were analyzed by SDS-PAGE and immunoblotted for β_3_ integrin. The immunoblotting bands were quantified by densitometry, and the data were normalized with respect to the controls.

Based on the above results, we were interested to discover if c-Abl kinase is involved in the signal pathway from PDGF receptor to α_v_β_3_ integrin. Serum-starved A375 cells were stimulated with PDGF for indicated time and the treated cells were lysed, then the lysates were used for coimmunoprecipitation. As shown in Fig. 4A, after PDGF stimulation, c-Abl kinase was present in the α_v_β_3_ integrin immunoprecipitated complexes, and the highest level of c-Abl kinase was detected after 10 min of stimulation. The association of α_v_β_3_ integrin and c-Abl kinase was dramatically blocked by STI571 incubation. Additionally, α_v_β_3_ integrin was detected in the c-Abl kinase immunoprecipitated complexes, the levels of coimmunoprecipitation changed in a time-dependent manner, and STI571 incubation reduced the association of α_v_β_3_ integrin and c-Abl kinase remarkably (Fig. 4B). To further confirm the association of α_v_β_3_ integrin and c-Abl kinase dependency on c-Abl kinase activity, we examined the phosphorylation status of the endogenous c-Abl kinase in response to PDGF stimulation. The results showed that the level of c-Abl kinase phosphorylation was increased within 10 min of PDGF stimulation, and the increase was partially inhibited by STI571 incubation (Fig. 4C). We next tested the endogenous c-Abl kinase activity by analyzing the phosphorylation status of Crk which is the endogenous substrate of c-Abl kinase. Indeed, the level of Crk phosphorylation was increased within 10 min of PDGF stimulation. When A375 cells were stimulated with PDGF attend by STI571, we found that STI571 inhibited the Crk phosphorylation induced by PDGF (Fig. 4D). To determine which domain of c-Abl kinase is responsible for the interaction between α_v_β_3_ integrin and c-Abl kinase, we prepared the GST-c-Abl SH3, GST-c-Abl SH2, GST-c-Abl SH3-2 and GST-c-Abl C-term fusion proteins (Fig. 4E). As shown in Fig. 4F, α_v_β_3_ integrin was found in GST-c-Abl SH3 and GST-c-Abl SH3-2, but not GST, GST-c-Abl SH2 or GST-c-Abl C-term pull-down complexes, and GST-c-Abl-SH3 and GST-c-Abl-SH3-2 bound equally to α_v_β_3_ integrin. The results above suggest that the activated c-Abl kinase interacts with the cytoplasmic domain of α_v_β_3_ integrin via its SH3 domain.

### The association of c-Abl kinase with α_v_β_3_ integrin is independent of cell adhesion and F-actin

**Figure 5:**
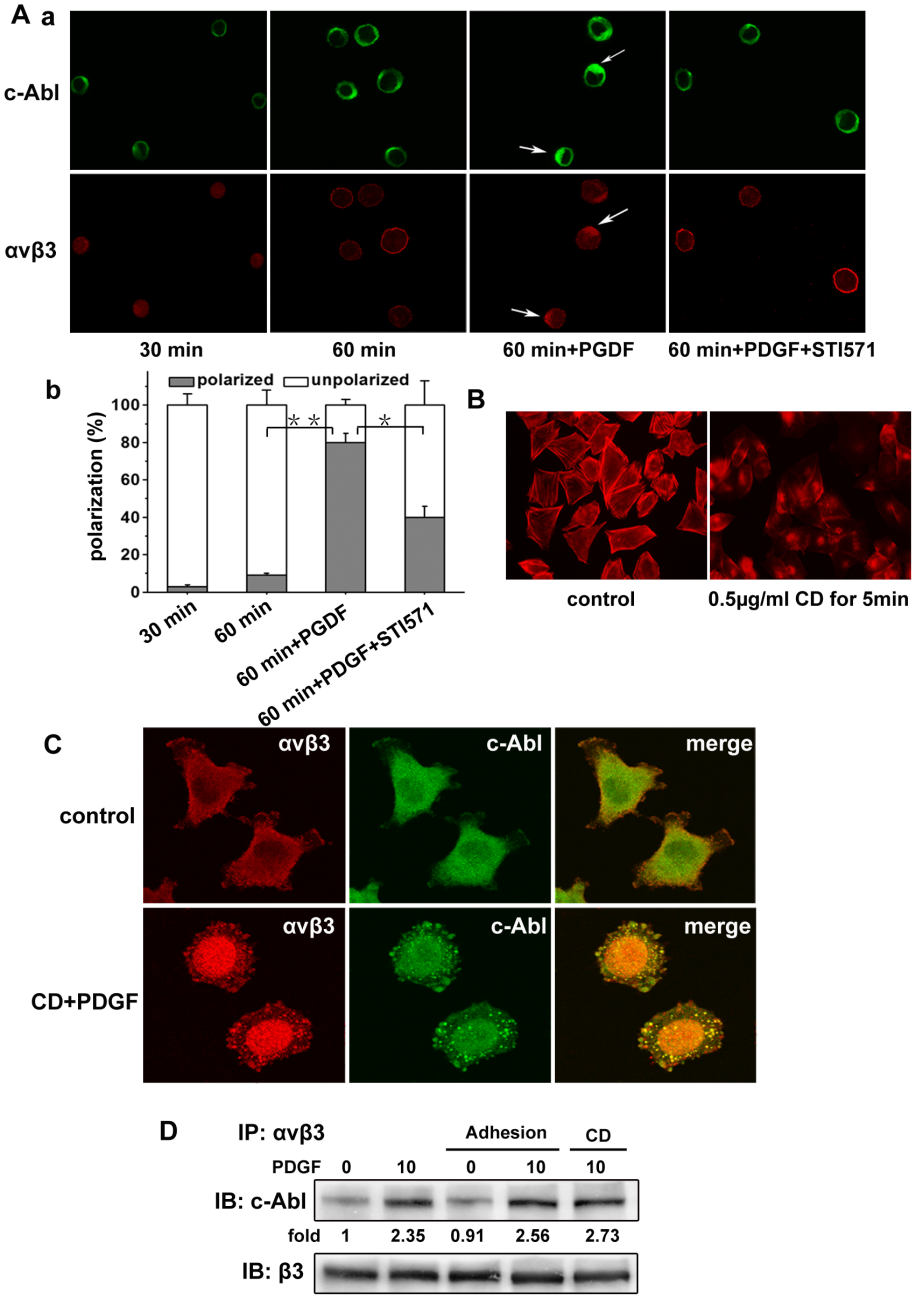
β_3_ integrin engagement and F-actin do not affect the interaction of c-Abl kinase and α_v_β_3_ integrin. (A) a. Serum-starved A375 cells were digested with EDTA and suspended in serum-free medium for 2 h to restore the state of the cells. After that the cells were incubated on fibronectin-coated coverslips with or without PDGF stimulation for indicated times. Then, the cells were fixed and stained for c-Abl kinase and α_v_β_3_ integrin. In inhibition experiments, cells were preincubated with STI571 for 10 min, and the inhibitor remained in the medium throughout the assay period. b. A375 cells were counted in 5–10 fields, and the percentages were calculated by dividing the number of polarized or unpolarized cells by the total number. Data were obtained from >25 cells for each field. (B) Cells were allowed to adhere and spread for 24 h, and Cytochalasin D (0.5 µg/ml) was added into the medium for 5 min. Then the cells were fixed and stained with phalloidin. (C) Serum-starved A375 cells were treated with Cytochalasin D (0.5 µg/ml) for 5 min and washed for three times with PBS. After stimulation with PDGF for 1 h, the cells were fixed and labeled with the antibodies of c-Abl kinase and α_v_β_3_ integrin. (D) Serum-starved A375 cells in suspension, as indicated, were stimulated with PDGF or allowed to adhere for 1 h, or treated with Cytochalasin D (0.5 µg/ml) for 5 min. Thereafter, the cells were lysed and the supernatants were incubated overnight at 4°C with α_v_β_3_ integrin antibody for immunoprecipitation. Immunoprecipitated complexes were collected with protein A-Sepharose beads, then were washed and resolved by SDS-PAGE and immunoblotted with indicated antibodies. Data are representative of three independent experiments.

Integrins function in bidirectional signaling, to further assess the molecular mechanism of the interaction between c-Abl kinase and α_v_β_3_ integrin, we investigated whether c-Abl kinase was involved in the outside-in signaling. As shown in Fig. 5A, serum-starved A375 cells were detached and replated on fibronectin-coated coverslips, and then the cells were allowed to adhere for 30 min and 60 min respectively. Confocal immunofluorescence microscopy showed that c-Abl kinase and α_v_β_3_ integrin were approximately uniform in the cytosol after cell adhesion for 30 min or 60 min. However, in the presence of PDGF, c-Abl kinase was distributed and colocalized with α_v_β_3_ integrin, and STI571 could inhibit the polarization of c-Abl kinase and α_v_β_3_ integrin. Here our results show that the binding of α_v_β_3_ integrin to ligands (cell adhesion) cannot affect the association of c-Abl kinase with α_v_β_3_ integrin. The redistributions of c-Abl kinase and α_v_β_3_ integrin were produced by the PDGF stimuli, and the redistribution of c-Abl kinase affect the redistribution of α_v_β_3_ integrin.

c-Abl kinase plays important roles in F-actin dynamics, yet its role in growth-factor-induced cell motility is not well defined [13]. Considering c-Abl kinase can bind to F-actin, we next asked whether c-Abl kinase associates with α_v_β_3_ integrin via F-actin. We first treated the spreading A375 cells with Cytochalasin D to inhibit the function of F-actin at different concentrations and time frames. We found that Cytochalasin D could significantly reduce the quantity of F-actin at concentration of 0.5 µg/ml for 5 min and failed to make the cell detachable (Fig. 5B). To evaluate the impact of F-actin on the interaction between c-Abl kinase and α_v_β_3_ integrin, A375 cells were treated with Cytochalasin D using the above concentration and time. Then the cells were stimulated with PDGF, and labeled with the antibodies of c-Abl kinase and α_v_β_3_ integrin respectively. As shown in Fig. 5C, c-Abl kinase and α_v_β_3_ integrin were highly colocalized and formed clustered spots in the cells. To further investigate the impact of cell adhesion and F-actin on the association of c-Abl kinase with α_v_β_3_ integrin, we examined the interaction of c-Abl kinase and α_v_β_3_ integrin in A375 cells after cell adhesion or pretreatment with Cytochalasin D by coimmunopreciptation assay. As shown in Fig. 5D, consistent with the above results, ligand binding to α_v_β_3_ integrin (cell adhesion) failed to increase the association between c-Abl kinase and α_v_β_3_ integrin. Also, inhibiting the de novo actin polymerization by Cytochalasin D failed to decrease the association between c-Abl kinase and α_v_β_3_ integrin. These results strongly suggest that the association of c-Abl kinase with α_v_β_3_ integrin is cell adhesion-independent and F-actin is not required for this association in A375 cells.

### c-Abl kinase regulates α_v_β_3_ integrin via talin

**Figure 6:**
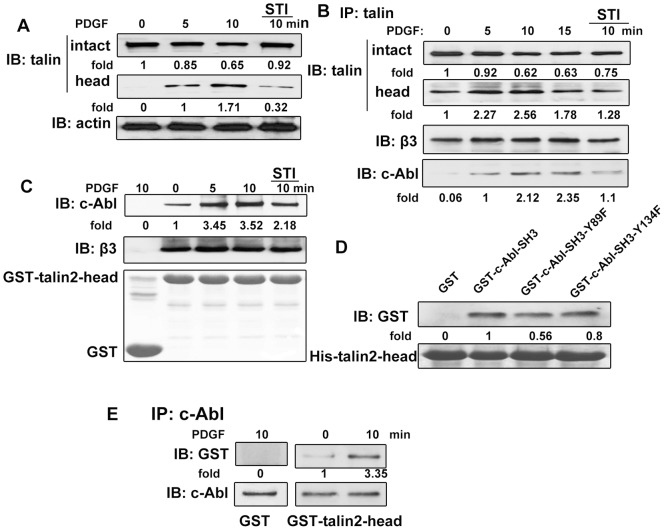
c-Abl kinase directly associates with talin head domain and regulates β_3_ integrin ligation concomitantly after PDGF stimulation. (A) Overnight serum-starved A375 cells were either untreated or treated with 20 ng/ml PDGF for the indicated times. Thereafter, the cells were lysed, and the lysates were immunoblotted with indicated antibodies. In inhibition experiments, cells were preincubated with STI571 for 10 min, and the inhibitor remained in the medium throughout the assay period. (B) Serum-starved A375 cells were stimulated as above. The lysates were immunoprecipitated with anti-talin antibody and immunoblotted with indicated antibodies. (C) Serum-starved A375 cells were treated as above. The lysates were incubated with GST or GST fusion proteins, and the bound proteins were separated by SDS-PAGE and immunoblotted with indicated antibodies. (D) GST or GST fusion proteins were incubated with Ni^2+^Sepharose coated with His-talin2-head. Bound proteins were separated by SDS-PAGE and immunoblotted with anti-GST antibody. (E) c-Abl kinase immunoprecipitates from PDGF treated or untreated A375 cells were separated by SDS-PAGE and transferred onto nitrocellulose membrane. After incubation with GST or GST-talin2-head, the membrane was immunoblotted with anti-GST antibody. Blots were immunoblotted with c-Abl kinase antibody to show equal loading. The immunoblotting bands were quantified by densitometry, and data were normalized with respect to the controls. Data are representative of three independent experiments.

The results above showed that c-Abl kinase interacts with the cytoplasmic domain of β_3_ integrin after PDGF stimulation, and a Far western blot analysis revealed that this interaction was not direct (data not shown). In addition, this interaction was not due to the cell adhesion and F-actin. It is unclear how c-Abl uses its SH3 domain to interact with α_v_β_3_ integrin. One possibility is that other proteins are required for c-Abl kinase to interact with α_v_β_3_ integrin. Talin is a major cytoskeletal protein which has an N-terminal head region of 50 kDa and an elongated helical rod of 220 kDa. Talin can bind to the cytoplasmic domains of β-integrin with its head domain. Moreover, the binding of talin to β-integrin triggers a conformational change in the integrin extracellular domain which increases its affinity for ECM proteins [9]. We first detected the existent state of talin in serum-starved A375 cells in response to PDGF stimulation. Starved cells or stimulated cells were lysed and then separated by SDS-PAGE and immunoblotted with talin head domain antibody TA205. As shown in Fig. 6A, there was only the intact 270 kDa talin in the serum-starved cells. When cells were stimulated with PDGF, talin underwent proteolysis to generate a 50 kDa head, which increased with the stimulation prolongation. Pretreating cells with STI571 partially inhibited the generation of talin head. We also investigated the association of talin, β_3_ integrin and c-Abl kinase in A375 cells by coimmunoprecipitation assay. As shown in Fig. 6B, the intact 270 KDa talin was cleaved to generate the 50 kDa fragment in response to PDGF stimulation, and c-Abl kinase was present in the immunoprecipitated complexes of talin after PDGF stimulation. As a point of emphasis, the change in amount of c-Abl kinase was consistent with the generation of talin head. In addition, as expected, talin and β_3_ integrin were constitutively present in the same immunoprecipitated complexes. As shown in Fig. 6C, a pull-down assay by using GST- talin2-head also confirmed these interactions. c-Abl was found in the GST- talin2-head pull-down complexes and the amount increased with PDGF stimulation prolongation. The inhibition of c-Abl kinase activity reduced the interaction of talin head and c-Abl kinase. Meanwhile, β_3_ integrin was constitutively associated with talin head, and this association was not affected by PDGF stimulation. These results indicate that PDGF stimulation can induce the proteolysis of intact talin, and the phosphorylated c-Abl kinase interacts with talin head which constitutively associated with β_3_ integrin.

To further reveal the mechanism of the interaction between c-Abl kinase and talin, we performed in vitro protein binding assay. Our above studies indicated that c-Abl kinase interacted with the cytoplasmic domain of β_3_ integrin via its SH3 domain. There are tyrosine residue Y89 and Y134 in the SH3 domain of c-Abl kinase. To test whether these sites were responsible for the association of talin head and c-Abl kinase, GST-c-Abl-SH3 Y89F and Y134F were used for pull down assays. As shown in Fig. 6D, Y134F and Y89F both reduced the binding of talin to the SH3 domain of c-Abl kinase. To detect whether talin head domain directly binds to c-Abl kinase, a Far western blot analysis was performed. The results showed that GST-talin2-head directly interacts with c-Abl kinase (Fig. 6E). Thus, talin head was found to interact directly with c-Abl kinase and function in the activation of c-Abl kinase.

## Discussion

The molecular mechanisms that induce cell migration are complex and involve the interplay of numerous receptors, including growth factor receptors and integrins, and their associated signaling intermediates. In this paper, we uncover a new function of c-Abl kinase in melanoma cell migration mediated by α_v_β_3_ integrin after activation of PDGF receptor. c-Abl kinase was mainly involved in the inside-out signaling of α_v_β_3_ integrin and potentiated the biological activity of α_v_β_3_ integrin. On the contrary, c-Abl kinase exhibited low sensitivity to outside-in (ligand-binding) signaling of α_v_β_3_ integrin. c-Abl kinase was activated by PDGF and associated with β_3_ integrin cytoplasmic domain via its SH3 domain, and this association was insusceptible to F-actin, but c-Abl kinase did not interact with β_3_ integrin directly. Talin head domain constitutively interacted with β_3_ integrin cytoplasmic domain and linked c-Abl kinase and β_3_ integrin together.

Earlier reports demonstrated that c-Abl kinase has a critical role in the migration of human liver cells and NIH3T3 cells [20]. However, little is known if c-Abl kinase functions in cancer cell migration. We found that c-Abl kinase was involved in A375 cell migration induced by PDGF. This finding is supported adequately by a series of experiments. First, we found c-Abl kinase inhibitor STI571 suppresses A375 cell migration. The fact that c-Abl kinase is involved in tumor cell migration was also reflected in the transfected cells: targeting c-Abl with its specific siRNA inhibited A375 cell migration, and *vice versa*, the overexpression of wild-type c-Abl kinase increased A375 cell migration.

As we know, integrin plays a crucial role in tumor cell migration, especially β_1_ and β_3_-integrins [8]. Using flow cytometry, we showed that α_v_β_3_ and β_1_ integrin were expressed on A375 cell surface, but the antibody blocking experiments revealed that the PDGF induced A375 cell migration was mainly mediated by integrin α_v_β_3_ but not β_1_. This may be due to the facts that PDGF cause α_V_β_3_ integrin activation but not β_1_ integrin and the phosphorylated PDGF receptor is associated with α_V_β_3_ integrin but not with β_1_ integrins [28]–[30]. Next we wanted to know if c-Abl kinase had any relationship with α_v_β_3_ integrin during cell migration. Our microscopy data demonstrated that, in the serum-starved A375 cell, endogenous c-Abl kinase and α_v_β_3_ integrin were co-localized dynamically in response to PDGF stimulation, and c-Abl kinase and α_v_β_3_ integrin formed clustered spots when cells were stimulated with PDGF for 60 min. These data imply that c-Abl kinase possesses a potential effect on regulating α_v_β_3_ integrin localization. For the function of integrin, conformational change and clustering are both likely to be important. Changes in the conformation of individual integrin into clustering and oligomers can influence the binding of its ligands. During the process, the integrin affinity and avidity need to be regulated from a low to a high state [31]. Our results in adhesion assay showed that the PDGF stimulation enhances the binding of integrin to its ligands and inhibition of c-Abl kinase affects the integrin binding activity (Fig. 3C). After PDGF stimulation, c-Abl kinase interacted with β_3_ integrin, and the interaction between c-Abl kinase and β_3_ integrin was attenuated after PDGF stimulation for 10 min. It is possible that c-Abl kinase phosphorylation was a transient process and the downstream activation of c-Abl kinase may serve as a negative feedback regulation to limit the duration of c-Abl activation, as previously suggested [32]. By using in vivo and in vitro kinase assays, we verified that PDGF stimulation increased c-Abl kinase activity within 10 min (Fig. 4), implying that kinase activity was required for c-Abl kinase to interact with β_3_ integrin. The SH3 and SH2 domains of c-Abl kinase are thought to function by mediating the interaction between c-Abl kinase and its substrates [33]. The SH3 domain preferentially interacts with the proteins containing a proline-rich region to inhibit c-Abl kinase activity [34]. Here, we find that the β_3_ integrin interacts with the SH3 domain of c-Abl kinase in A375 cells, but this interaction is not direct (Fig. 4E).

As a cytoplasmic tyrosine kinase, c-Abl is mainly linked to the transduction of extracellular signals through interaction with cell surface receptors. In our previous study, c-Abl kinase was shown to be involved in β_2_ integrin outside-in signaling in neutrophil [35]. Others reported that in fibroblasts, c-Abl kinase activation occurred after integrin-mediated cell attachment to fibronectin [21]. Here our results show that the binding of α_v_β_3_ integrin to ligands cannot affect c-Abl kinase redistribution and its association with α_v_β_3_ integrin (Fig. 5). Thus, c-Abl kinase was not involved in the outside-in signaling of α_v_β_3_ integrin. It became clear that most leukocyte integrin (β_2_) and platelet integrin (α_IIb_β_3_), including β_1_-containing integrins, exist in a resting state until activated by stimulation. Whereas α_V_β_3_ integrin is constitutively active on many cell types. Studies have been shown that the interactions between leg domains are strong for α_IIb_β_3_, but the association between α_V_ and β_3_ is weak, which may help to explain why the default state of α_IIb_β_3_ integrin on platelets is completely inactive and α_V_β_3_ integrin is constitutively active [9]. We suggest that the constitutive activation of integrin failed to arouse c-Abl kinase activation. Collectively, c-Abl kinase functions in different integrin signaling in various manners.

There have been reports that c-Abl kinase can be activated by various stimuli and the activated c-Abl kinase has been shown to activate many cellular proteins to regulate cytoskeletal dynamics [17], [36]. The linkage between integrin and the actin cytoskeleton appears to be crucial for the integrin function [37]. We also asked if c-Abl kinase could regulate α_V_β_3_ integrin via actin. In CD treated cells, we noticed that c-Abl kinase still co-localizes with α_V_β_3_ integrin after PDGF stimulation, and CD did not attenuate the interaction of c-Abl kinase and α_V_β_3_ integrin. So, we have provided the evidence that c-Abl kinase regulates α_V_β_3_ integrin clustering independent of actin (Fig. 5), and we believe there exist some other proteins close to α_V_β_3_ integrin that assisted c-Abl kinase to regulate α_V_β_3_ integrin. To the best of our knowledge, talin is one of few proteins directly linked to integrin cytoplasmic tail. In vertebrates, there are two talin genes (talin1 and talin2) that encode closely related proteins (74% identity). The intact talin protein (270 kDa) can be proteolysed by proteases such as calpain and trypsin to generate an N-terminal 50-kDa head domain and a 220-kDa C-terminal rod domain [38], [39]. The head domain binds to the integrin cytoplasmic domain and is required for integrin activation. In our studies, we found that talin was cleaved into head domain and rod domain after PDGF stimulation (Fig. 6A). Although talin was cleaved after PDGF stimulation, the amount of β_3_ integrin associated with talin head domain (include intact talin) were not affected by PDGF stimulation, whereas c-Abl kinase only interacted with cleaved talin head domain (Fig. 6B–C). So, we think that talin constitutively and directly associates with the cytoplasmic domain of β_3_ integrin in A375 cells, which may be partially due to the constitutively active α_V_β_3_ integrin with low affinity. When cells were stimulated with PDGF, the local concentration of active talin increased, the cellular environment regulated the local complex formation that made integrin clustering and then the integrin affinity became higher. Since the formation of a precise complex between the β-tail of integrin, talin and the membrane is the final key step in inside-out activation of α_V_β_3_ integrin [40], it is important to mention that c-Abl kinase plays a necessary role in regulating talin activity. We found c-Abl kinase inhibitor STI571 suppressed talin activity *in vivo* and *in vitro* (Fig. 6A–C). Thus, we consider that it is possible that c-Abl kinase is an upstream regulator of talin in α_V_β_3_ integrin signaling in response to PDGF stimulation in A375 cell. In addition, our results show that c-Abl kinase directly associates with talin head domain (Fig. 6E), both the Y89F and Y134F mutants of c-Abl SH3 domain partially reduced the binding of talin head (Fig. 6D), indicating that these two sites are both important but neither one is unique.

The fact that growth factor receptors regulating integrin inside-out signals derived from G-protein, phosphatidylinositol-4,5-bisphosphate (PtdIns(4,5)P2) hydrolysis and PKC has been confirmed. However, the specific mechanisms involved are still under investigation [41]. Adding to the possible oncogenic roles of c-Abl kinase in both NSCLC and breast cancer. As we mentioned above, our studies have provided new insights into the nature of the crosstalk between growth factor receptors and integrin by measuring the novel role of c-Abl kinase in regulating melanoma cell migration: c-Abl kinase is critical for the formation of early clusters of integrin complex, and the complex might be the sites of PDGF receptor activation in the early stage of cell spreading in a new cycle of motile melanoma cells. However, the signaling pathways that c-Abl kinase regulate talin activity are only just beginning to emerge, and the structural basis for the activation of many ligand-binding sites in talin is not yet fully understood.
